# Ability of an altered functional coupling between resting-state networks to predict behavioral outcomes in subcortical ischemic stroke: A longitudinal study

**DOI:** 10.3389/fnagi.2022.933567

**Published:** 2022-09-15

**Authors:** Yongxin Li, Zeyun Yu, Ping Wu, Jiaxu Chen

**Affiliations:** ^1^Guangzhou Key Laboratory of Formula-Pattern of Traditional Chinese Medicine, Formula-Pattern Research Center, School of Traditional Chinese Medicine, Jinan University, Guangzhou, China; ^2^Acupuncture and Tuina School/Third Teaching Hospital, Chengdu University of Traditional Chinese Medicine, Chengdu, China

**Keywords:** functional MRI, subcortical stroke, independent component analysis, functional network connectivity, recovery prediction

## Abstract

Stroke can be viewed as an acute disruption of an individual’s connectome caused by a focal or widespread loss of blood flow. Although individuals exhibit connectivity changes in multiple functional networks after stroke, the neural mechanisms that underlie the longitudinal reorganization of the connectivity patterns are still unclear. The study aimed to determine whether brain network connectivity patterns after stroke can predict longitudinal behavioral outcomes. Nineteen patients with stroke with subcortical lesions underwent two sessions of resting-state functional magnetic resonance imaging scanning at a 1-month interval. By independent component analysis, the functional connectivity within and between multiple brain networks (including the default mode network, the dorsal attention network, the limbic network, the visual network, and the frontoparietal network) was disrupted after stroke and partial recovery at the second time point. Additionally, regression analyses revealed that the connectivity between the limbic and dorsal attention networks at the first time point showed sufficient reliability in predicting the clinical scores (Fugl-Meyer Assessment and Neurological Deficit Scores) at the second time point. The overall findings suggest that functional coupling between the dorsal attention and limbic networks after stroke can be regarded as a biomarker to predict longitudinal clinical outcomes in motor function and the degree of neurological functional deficit. Overall, the present study provided a novel opportunity to improve prognostic ability after subcortical strokes.

## Introduction

Stroke is one of the most frequent causes of chronic disability worldwide. Unlike many other neurological disorders, a stroke appears suddenly in the brain system. Most stroke cases are ischemic or caused by a lack of blood supply to cerebral tissue due to embolic occlusion of a cerebral artery (Rehme and Grefkes, 2013). Aphasia and motor deficits are particularly prevalent long-term disabilities after stroke significantly impacting public health. Thus, understanding the neurobiological factors determining functional outcomes would offer insights into stroke treatment approaches. Functional magnetic resonance imaging (fMRI) can provide a non-invasive means to understand the neural mechanisms of brain reorganization after stroke (Ovadia-Caro et al., 2014).

Stroke can be viewed as an acute disruption of an individual’s connectome caused by a focal or widespread loss of blood flow. Patients with ischemic stroke showed functional and structural reorganization of perilesional and remote brain regions. Using a connectivity-based approach can provide unique opportunities to study the effect of a stroke on brain recovery (Grefkes and Fink, 2014). Previous studies reported disruptions in functional brain connections between distinctive cortical areas following strokes (Grefkes and Ward, 2014; Mirzaei and Adeli, 2016; Chen et al., 2021). For example, several studies on stroke demonstrated reduced functional connectivity between the bilateral motor regions (Carter et al., 2010, 2012a; Wang et al., 2010; Min et al., 2020). Abnormal interhemispheric functional connectivity patterns within the motor network were correlated with motor deficits after strokes (Baldassarre et al., 2016; Li et al., 2016). Although the effects of stroke on the functional networks of the brain were mainly focused on the motor network, patients with stroke also exhibited significant changes in functional connectivity in other subnetworks, such as the frontoparietal network (FPN) (Nomura et al., 2010; Hordacre et al., 2021), the default mode network (DMN) (Tuladhar et al., 2013; Jiang et al., 2018), the attention-related network (Zhao et al., 2018a), and the visual network (VIS) (Park et al., 2011). A previous neuroimaging study showed a double dissociation between an abnormal functional connectivity pattern and attention and motor deficits in patients with a right hemisphere stroke (Baldassarre et al., 2016). Abnormal patterns in the brain networks after a stroke can reflect the corresponding behavioral deficits. Additionally, changes in the internetwork functional connectivity of multiple networks following strokes were also investigated (Wang et al., 2014; Zhao et al., 2018b). Patients with stroke demonstrated alterations in both motor-visual networks and other high-order motor-cognitive networks. Together, these findings consistently demonstrate extensive changes in the functional network organization in patients with stroke.

Recently, the effects of the intervention on network organization in patients with stroke were also investigated to understand the recovery of motor or other cognitive functions (Bajaj et al., 2015; Zheng et al., 2016; Sinha et al., 2021). For example, a previous study on strokes revealed functional restoration of the brain’s motor-execution network after a combination of mental practice and physical therapy (Bajaj et al., 2015). Three weeks of upper limb rehabilitation therapy in patients with stroke can result in an increase in interhemispheric communication among motor areas, which is accompanied by improvements in behavioral performance (James et al., 2009). Our recent studies, which used a longitudinal design, revealed that motor-related networks and motor function in patients with subcortical stroke showed recovery after 1 month of clinical treatment (Li et al., 2016, 2017, 2021). However, all of these previous studies were focused only on the intervention effect of interactions between brain regions within a subnetwork; few studies used neuroimaging to detect longitudinal changes in interactions between networks during the recovery process in patients with stroke.

Thus, the present study aimed to examine the longitudinal changes in the functional organization within and between resting-state networks of the whole brain in patients with chronic subcortical stroke. For this purpose, we employed a method called independent component analysis (ICA) to construct functional brain networks. ICA is a data-driven approach for delineating spatially independent patterns of coherent signals (van de Ven et al., 2004). The interactions within and between multiple brain networks can be measured directly (Zhao et al., 2018b). Previous studies on certain neurological disorders, such as schizophrenia and depression, indicated that ICA can efficiently detect intra- and inter-network connectivity patterns (Salman et al., 2019; Maglanoc et al., 2020). A recent stroke study used this resting-state fMRI method to assess hypoperfusion (Hu et al., 2021). Until now, only a few studies used the ICA method to investigate both intra- and inter-network connectivity changes in functional deficits after strokes (Wang et al., 2014; Zhao et al., 2018b; Chen et al., 2019). These previous studies used ICA to detect functional connectivity alterations within and between multiple networks in patients with stroke, but longitudinal changes in the organization of the brain were not investigated. In the present study, we applied the ICA method to resting-state fMRI data to investigate the functional recovery process within and between resting-state networks in stroke. Because stroke symptoms may reflect dysfunction across multiple systems, changes in the functional pattern of the brain after strokes have important implications for cognitive recovery (Carter et al., 2012b; Grefkes and Fink, 2020). We hypothesized the following: (1) disrupted functional organization within and between functional networks would be detected in patients with subcortical stroke, (2) disrupted functional organization in patients would be restored after a period of time, and (3) functional connectivity between networks in patients with stroke would reflect the patients’ clinical recovery.

## Materials and methods

### Subjects

We collected 21 subcortical adult patients with stroke from the Department of Neurology of the First Affiliated Hospital of Chengdu University of Traditional Chinese Medicine in China and 17 healthy controls in the present study. The present study was designed and carried out a few years ago. The sample size selection was referred to by the previous studies (Lindenberg et al., 2012; Wang et al., 2012, 2014), in which the general sample size of subjects in these previous neuroimaging studies of each group was from 15 to 30. We discarded data of two participants (one patient and one control) because of incomplete imaging data. Data of one patient were discarded because of head movement during the MRI scan. Therefore, 19 adult patients with stroke (eight women; mean age ± SD: 64.74 ± 12.42 years) were included in the final analysis. All patients were diagnosed with unimanual motor deficits due to subcortical ischemic lesions. Patients were included in the study based on the following criteria: (1) first-ever ischemic stroke with right-handedness before stroke; (2) strictly subcortical lesions and absence of other white matter pathology as verified by structural MRI; (3) time interval of at least 3 weeks between stroke onset and the time of study enrollment; (4) no additional psychiatric or neurological disorders; (5) absence of neglect, aphasia, and dementia; and (6) no subsequent cerebral ischemia. None of the patients had undergone any other experimental therapy before enrollment in this study. Resting-state fMRI data of all patients were collected at two time points: before and 1 month after the antiplatelet therapy. Clinical scores were assessed for these patients at the above two time points. Sixteen healthy subjects (six women, mean age ± SD: 64.75 ± 10.51 years) without any history of neurological or psychiatric disorders were included in the final analysis. All of the control subjects were right-handed. The participants in the control group were well matched to the patients with stroke with regard to age, gender, and handedness. All control subjects were scanned only one time, on the day they were recruited. Table 1 provides detailed information on both groups. The protocol was approved by the Ethics Committee of Chengdu University of Traditional Chinese Medicine (no. 2011KL-002), and the research was carried out in accordance with the Declaration of Helsinki. Written informed consent was obtained from each subject prior to the study.

**TABLE 1:** Demographic and clinical information data of the subjects.

Characteristics	Patient group (*n* = 19, Mean ± SD)		Control group (*n* = 16, Mean ± SD)	Statistics	*P*-value
Gender (men/women)	11/8		10/6	χ^2^(1) = 0.077	0.782
Age (ages)	64.74 ± 12.42		64.75 ± 10.51	*t*(33) = –0.003	0.997
Duration of illness (days)	55.53 ± 46.75		\	\	\
Lesion sizes (cm^3^)	0.85 ± 0.19		\	\	\
FMA_pre_post	Pre: 84.26 ± 4.05	Post: 91.53 ± 4.06	\	*t*(18) = –10.08	0.000
NDS_pre_post	Pre: 23.26 ± 4.28	Post: 14.21 ± 5.17	\	*t*(18) = 12.96	0.000

Summary values are reported as the means ± standard deviation. Duration of illness refers to how long the patients were enrolled after the stroke. FMA, Fugl-Meyer Motor Assessment; NDS, neurological deficit scores.

### Clinical assessments

Antiplatelet therapy was administered to all the patients (75 mg of clopidogrel one time each day taken orally and 10 mg of Erigeron breviscapus injection). Additionally, citicoline (0.5 g, daily) was injected intravenously to improve the clinical outcome following ischemic stroke. Drug therapy was conducted for 1 month (30 days) for each patient. Information on the Fugl-Meyer Assessment (FMA) and the Neurological Deficit Scores (NDS) was assessed two times from all patients on the days the imaging data were collected. FMA has been widely used to evaluate the motor functions of patients with stroke (Gladstone et al., 2002). Higher FMA scores indicate milder impairments in motor function. NDS constitutes an observational assessment of the severity of neurological functional deficits and stroke severity. This score resembles the NIHSS score, which is used in clinical practice. A paired *t*-test was conducted to determine whether the clinical scores of the patients with stroke had changed between the two time points.

### Image acquisition

All images were obtained using a 3T Siemens scanner (MAGNETOM Trio Tim, Siemens, Germany) at the West China Hospital MRI Center, Chengdu, China. During the scanning process, foam cushions were used to reduce head movements and scanner noise. Whole-brain resting-state fMRI was performed using an echo-planar imaging (EPI) sequence: 30 interleaved axial slices; slice thickness = 5 mm; matrix = 64 × 64; repetition time = 2 s; echo time = 30 ms; flip angle = 90°; field-of-view (FOV) = 240 mm × 240 mm; and 180 volume; three-dimensional T1-weighted structural MRI was performed using a spin-echo planar image sequence with the following parameters: repetition time/echo time = 1,900 ms/2.26 ms; flip angle = 9°; in-plane matrix resolution = 256 × 256; slices = 176; field of view = 256 mm × 256 mm; and voxel size = 1 mm × 1 mm × 1 mm. The head coil and foam cushions were used during scanning to reduce head movement. During the acquisition of the imaging data, the participants were instructed to remain awake, remain motionless, keep their eyes closed, and try not to think about anything in particular.

### Lesion mapping

The lesion location for each patient on the T1-weighted MRI images was determined by one experienced neuroradiologist. We manually outlined the lesion profiles on the T1-weighted MRI images slice by slice using MRIcron software, and we generated a lesion mask for each patient. Then, for each patient, the T1 structural image was normalized to the MNI template, and the resulting parameter file was used to normalize the lesion mask. After spatial normalization, the lesion mask of each patient was normalized to MNI spaces (see Supplementary Figure 1A and Supplementary Table 1). The individual masks were used to evaluate the lesion volume of each patient. To ensure that all lesions were on the same hemisphere, the imaging data of two patients who had lesions on the right hemisphere were flipped from right to left along the midsagittal line. Additionally, the imaging data from the control subject matched the two patients who were midsagittally oriented. The union of all individual lesion masks was used to construct a group lesion map for the patients (see Supplementary Figure 1B). This overlapped lesion map helped us to see all patients’ lesion distribution.

### Imaging processing and group-independent component analysis

The resting-state fMRI data were processed using the Statistical Parametric Mapping (SPM8^1^) package. The preprocessing steps included slice timing, spatial realignment, normalization into the Montreal Neurological Institute template, and smoothing (6 mm FWHM). Slice timing and head motion correction were performed, and a mean functional image was obtained for each participant. The excessive motion was defined as more than 3 mm of translation or greater than a 3° rotation in any direction. The mean framewise displacement (FD) was computed by averaging the FD of each participant across the time points. The participants were excluded if the mean FD was less than 0.5 mm or more than 20% of all time points with FD values exceeding 0.5 mm. Only one patient was excluded due to excessive motion, and no participant was excluded due to excessive FD. Then, the mean FD values for the remaining participants were included for the comparison between the patients and healthy subjects. No significant differences were found between groups of FD (Stroke_1st: 0.19 ± 0.07, Stroke_2nd: 0.20 ± 0.08, Controls: 0.19 ± 0.11, *F* = 0.088, *p* = 0.916). Each participant’s T1-weighted structural image was co-registered to their mean functional image and then segmented. The functional images were then normalized to the standard Montreal Neurological Institute space using the T1 image unified segmentation; they were then resampled to 3 mm and smoothed using a 6-mm full-width at half maximum Gaussian smoothing kernel. The preprocessed resting state data of all of the participants were analyzed using ICA as implemented in the GIFT software^2^ (Calhoun et al., 2001). Spatial ICA analysis is a data-driven approach performed using a group ICA in the GIFT. This method extracts the non-overlapping spatial maps with temporally coherent time courses that maximize independence. We used the standard procedure in GIFT. The preprocessed data from all participants were concatenated into a single dataset. Data reduction was conducted using principal component analysis to reduce the dimensions of the functional data. ICA was performed to decompose the grouped data into 28 independent components using an Infomax algorithm (Bell and Sejnowski, 1995). This step was repeated 20 times using the ICASSO algorithm to assess independent components’ repeatability or stability (ICs). Aggregate spatial maps were estimated as the modes of the component clusters. ICs and time courses for each participant were back-reconstructed.

A systematic procedure was used to diagnose the artifacts and identify the functional networks. We used the cortical parcelation maps of the Yeo2011 resting state network^3^ as a template and multiple linear regression, as implemented in the spatial sorting function of GIFT, to compare the spatial pattern of each IC with these templates. The functional “Component Labeller” module in the GIFT toolbox was used to produce a txt file containing a correlation index. Components with a higher correlation (larger than 0.2) with these maps of Yeo2011 templates were considered the most related components. The reason for selecting this correlation criterion is based on previous references, in which a reasonable choice of correlation value was studied between ICs from different datasets (Smith et al., 2009; Fu et al., 2019). Some components were excluded from the remainder of the analysis because they correlated with motion artifacts or spatial maps that included the white matter, the ventricular system, or the cerebral spinal fluid or because they had irregular time course spectral power. As a result, the potential components of interest (19 ICs) were identified by the above process.

### Functional network connectivity analysis

To detect the functional organization within and between functional networks after a stroke, functional network connectivity (FNC) analysis was performed. Before calculating the FNC between the time courses of the ICs, postprocessing procedures on the time courses were performed to remove the remaining noise sources, such as detrending linear, multiple regressions of the Friston 24 realignment parameters, and filtering (a bandpass filter of 0.01–0.1 Hz). Then, we calculated the FNC using the Pearson correlation coefficients between the time courses of the ICs. To normalize the variance in the correlation values, all of the resulting correlation coefficients were transformed into z-scores using Fisher’s *z*-transformation. The normalized correlation value of each pair was regarded as the network edges. An FNC matrix with a dimension of 19 × 19 was created for each subject. In the present study, these selected ICs were categorized into seven functional domains based on the Yeo2011 template: VIS, somatomotor network (SMN), dorsal attention network (DAN), ventral attention network (VAN), limbic network (LIM), FPN, and DMN (see Supplementary Figure 2A). Here, network connectivity was defined as the connectivity between these seven subnetworks (such as FNC between one IC of the DAN and one IC of the DMN). Network connectivity was defined as the connectivity among the components belonging to one subnetwork (such as the FNC between two ICs of the DMN).

We used a network-based statistic (NBS) approach for the functional connectivity networks to localize the specific connected components, which reflects the functional connections that differed between each pair of groups (Zalesky et al., 2010). A set of suprathreshold links among all the connected components was defined using the NBS method. The non-parametric permutation method was used to estimate the significance of each component (5000 permutations). Analyses were controlled for age, gender, duration of illness, and lesion size. The threshold (edge *p* < 0.05 and component *p* < 0.05) was adopted to address the multiple comparisons in the functional connectivity.

We further examined whether FNC changes after stroke can predict the recovery of clinical performance. This step focused on those functional connections between ICs with significant group differences. We performed linear regression analyses to determine those functional connections at the first time point to assess their ability to predict clinical performance (FMA and NDS) at the second time point. The regression model was considered significant at a *p*-value < 0.05. The Bonferroni method also corrected the regression results based on the pairs of ICs showing significant changes in FNC in patients with stroke. This process was performed using the SPSS statistical software (version 20.0, IBM Inc., Armonk, NY, United States).

## Results

### Behavioral data

No significant differences between the patients and the normal controls were observed in the sexes and ages (Table 1). Between the two time points of the patient group, the FMA score was significantly increased (paired *t*-test: *t* = –10.08, *p* < 0.001) from 84.26 ± 4.05 (first time point) to 91.53 ± 4.06 (second time point). The severity of the neurological functional deficit after stroke was assessed by the NDS and showed a significant decrease (pair T: *t* = 12.96, *p* < 0.001) from 23.26 ± 4.28 (first time point) to 14.21 ± 5.17 (second time point).

### Group independent component analysis and identification of potential interest components

**FIGURE 1:**
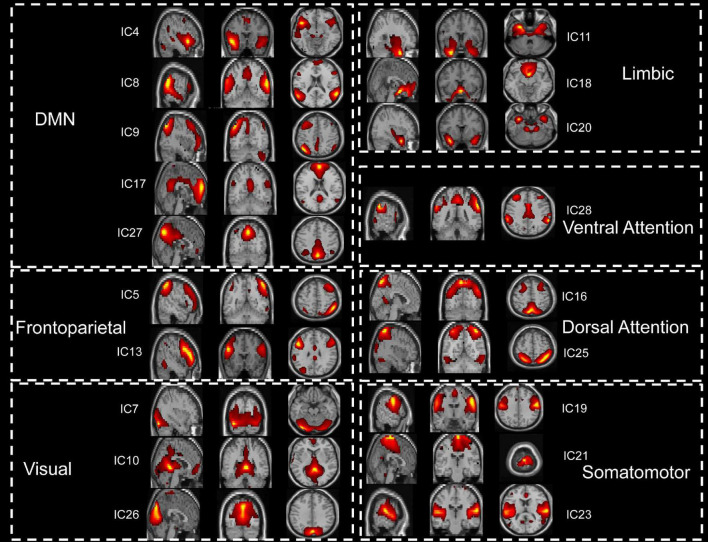
The 19 functionally relevant resting-state networks. IC, independent component; DMN, default mode network.

The spatial maps of potential interest components were identified for group ICA (Figure 1). Nine ICs were excluded, and nineteen potential components of interest were identified in group ICA. These selected ICs were categorized into seven functional domains: VIS, SMN, DAN, VAN, LIM, FPN, and DMN (see Supplementary Figure 2B).

### Between-group differences in functional network connectivity

**FIGURE 2:**
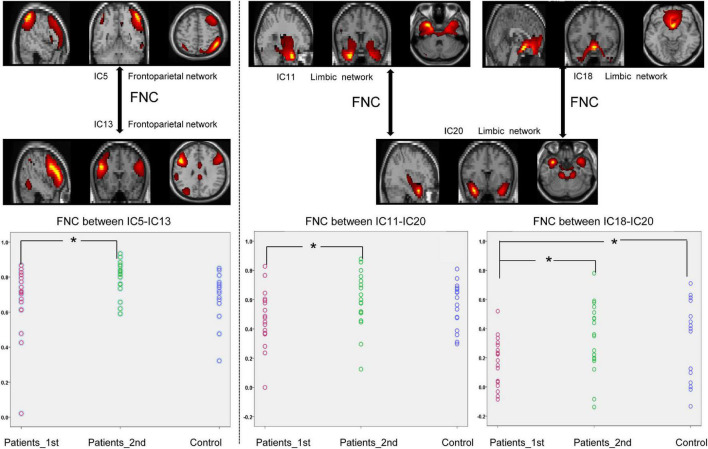
Significantly enhanced within-network connectivity of patients with subcortical ischemic stroke with a one-month follow-up. IC, independent component; Patients_1st, patients with stroke’ results at the first time point; Patients_2nd, patients with stroke’ results at the second time point. Significant changes in the FNC between groups: *p < 0.05.

The NBS approach was conducted for the functional connectivity networks between each pair of groups, and the statistical results demonstrated specific FNC alterations in patients with stroke. Paired *t*-test analysis of the patients with stroke demonstrated that the FNC within networks showed a significant increase at the second time point (Paired *t*-test, patients 1st vs. 2nd: FPN_IC5-FPN_IC13, *t* = –2.41, *p* = 0.021; LIM_IC11-LIM_IC20, *t* = –2.07, *p* = 0.045, LIM_IC18-LIM_IC20, *t* = –2.56, *p* = 0.014). The significant within-network changes in FNC in patients with stroke are shown in Figure 2.

**FIGURE 3:**
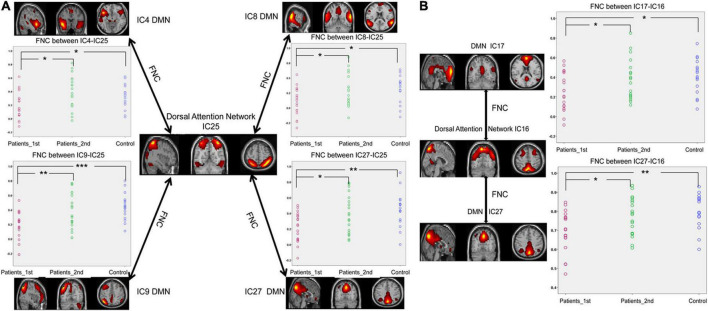
Significantly enhanced FNCs between the DAN and DMN in subcortical ischemic stroke patients with 1-month follow-up. **(A)** The FNCs between the DAN (IC25) and the DMN (IC4, IC8, IC9, IC27). **(B)** The FNCs between the DAN (IC16) and the DMN (IC17, IC27). IC, independent component; Patients_1st, stroke patients’ results at the first time point; Patients_2nd, stroke patients’ results at the second time point; DMN, default mode network. Significant changes in the FNC between groups: *p < 0.05; **p < 0.01; ***p < 0.001.

As displayed in Figure 3, the FNCs between the DMN and DAN showed a significant decrease in the patients with stroke compared with the controls (two sample *t*-test, 1st patient vs. control: IC4-IC25, *t* = –2.05, *p* = 0.046; IC8-IC25, *t* = –2.59, *p* = 0.014; IC9-IC25, *t* = –3.99, *p* = 0.0003; IC27-IC25, *t* = –3.15, *p* = 0.0035; IC17-IC16, *t* = –2.64, *p* = 0.012; IC27-IC16, *t* = –3.33, *p* = 0.0021). Significant increases in FNCs between the DMN and DAN were found from the 1st to the 2nd patients with stroke (Paired *t*-test, patients 1st vs. 2nd: IC4-IC25, *t* = –2.68, *p* = 0.011; IC8-IC25, *t* = –2.08, *p* = 0.045; IC9-IC25, *t* = –2.90, *p* = 0.006; IC27-IC25, *t* = –2.62, *p* = 0.013; IC17-IC16, *t* = –2.12, *p* = 0.041; IC27-IC16, *t* = –2.42, *p* = 0.021). No significant differences were detected in FNCs (DMN-DAN) between the controls and the patients with stroke at the second time point.

**FIGURE 4:**
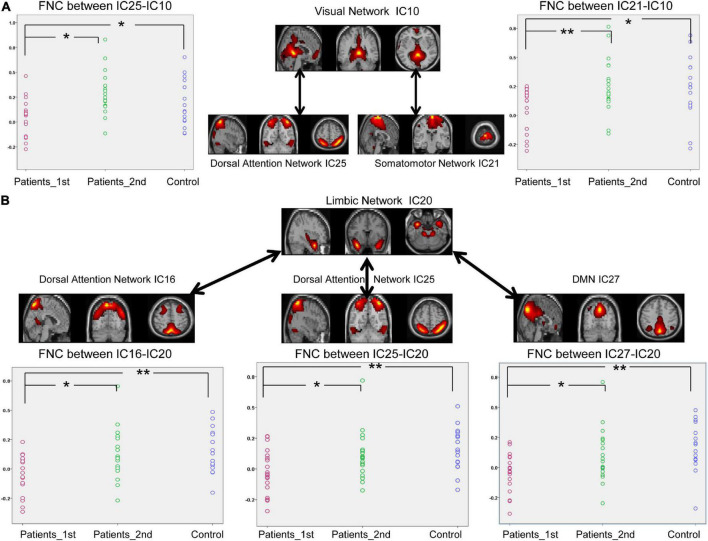
Significantly enhanced between-network connectivity of subcortical ischemic stroke patients with a 1-month follow-up. **(A)** The FNCs between the VIS (IC10) and the VAN (IC25) or between the VIS (IC10) and SMN (IC21). **(B)** The FNCs between the LIM (IC20) and the VAN (IC16, IC25) or between the LIM (IC20) and DMN (IC27). IC, independent component; Patients_1st, stroke patients’ results at the first time point; Patients_2nd, stroke patients’ results at the second time point. Significant changes of FNC between groups: *p < 0.05; **p < 0.01.

According to Figure 4, decreased FNCs between networks were also observed in the patients with stroke at the first time point (two sample *t*-test between 1st patient vs. control: DAN_IC25- VIS_IC10, *t* = –2.22, *p* = 0.033; SMN_IC21-VIS_IC10, *t* = –2.41, *p* = 0.021; DAN_IC16-LIM_IC20, *t* = –3.38, *p* = 0.0018; DAN_IC25-LIM_IC20, *t* = –2.96, *p* = 0.0056; DMN_IC27-LIM_IC20, *t* = –3.43, *p* = 0.0035). Paired *t*-test analysis of the patients with stroke revealed that the FNCs between these networks increased from the first to the second time point (Paired *t*-test, patients 1st vs. 2nd: DAN_IC25- VIS_IC10, *t* = –3.05, *p* = 0.0043; SMN_IC21-VIS_IC10, *t* = –2.88, *p* = 0.0067; DAN_IC16-LIM_IC20, *t* = –2.55, *p* = 0.015; DAN_IC25-LIM_IC20, *t* = –2.11, *p* = 0.042; DMN_IC27-LIM_IC20, *t* = –2.21, *p* = 0.034). There was no longer a significant difference in the FNCs between the controls and the patients at the second time point.

### Regression analyses to predict clinical scores

**FIGURE 5:**
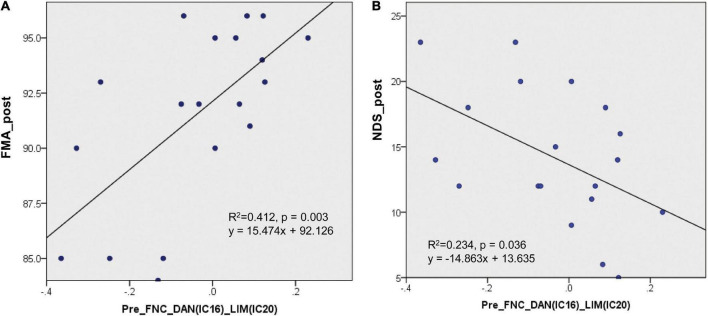
Regression results of the FNC at the first time point to predict the clinical assessment performance at the second time point. **(A)** The regression involving the FNC values between the DAN and LIM (IC16-IC20) at the first time point produced a predictive regression of the FMA values at the second time point (*R*^2^ = 0.412, *p* = 0.003). **(B)** The regression involving the FNC values between the DAN and LIM (IC16-IC20) at the first time point produced a predictive regression of the NDS values at the second time point (*R*^2^ = 0.234, *p* = 0.036).

In the present study, 14 pairs of ICs showed significant changes in the FNC in patients with stroke. The regression involving the FNC values between the DAN and the LIM (IC16-IC20) at the first time point produced a predictive regression of the FMA values at the second time point (*R*^2^ = 0.412, *p* = 0.003, see Figure 5A). This regression result (*p* = 0.003) could withstand the Bonferroni correction (threshold at 0.0036 = 0.05/14). At the same time, the regression involving the FNC values between the DAN and the LIM (IC16-IC20) at the first time point also produced a predictive regression of the NDS values at the second time point (*R*^2^ = 0.234, *p* = 0.036, see Figure 5B). This regression result could not withstand the Bonferroni correction (threshold at 0.0036 = 0.05/14).

## Discussion

The present study employed the FNC analysis to investigate the changes within and between brain networks with a longitudinal design. We further explored the potential predictive ability of functional recovery using functional coupling between resting-state networks after stroke. Our results demonstrated the following: (a) the FNCs within and between multiple brain networks were disrupted after strokes, (b) these disrupted FNCs were restored to a nearly normal level 1 month later, and (c) the FNC between the DAN and the LIM at the first time point yielded a predictive regression associated with the later clinical performance. These overall findings suggest that longitudinal changes in FNCs within and between networks can provide relevant information on brain recovery after stroke. The regression analyses suggest that internetwork functional coupling after stroke can predict the recovery of neurological functional ability.

### Altered functional network connectivity after subcortical strokes

Relative to the normal controls, the patients with stroke exhibited a significant decrease in the internetwork functional coupling between multiple networks (including the DAN, DMN, LIM, VIS, and SMN). Our results are consistent with previous studies that reported decreased functional connectivity within and between the resting state networks in patients with stroke (Wang et al., 2014; Bajaj et al., 2015; Baldassarre et al., 2016). Here, we found that patients with stroke exhibited decreased internetwork FNC between the DAN and DMN. The DAN is an identified functional brain network that includes regions in the posterior parietal cortex, the frontal eye fields, and the visual area of MT + (Corbetta and Shulman, 2002). Previous studies showed that this subnetwork is related to unilateral spatial neglect (Carter et al., 2010). A previous study in patients with stroke demonstrated a significant decrease in the interhemispheric functional connectivity in the DAN and motor networks (Baldassarre et al., 2016). The DMN is an important resting-state functional network of the brain and is composed of a set of functionally highly connected regions (Raichle et al., 2001). Previous resting-state functional studies on patients with stroke commonly observed DMN disruption (Tuladhar et al., 2013). Resting-state functional connectivity impairments of the DMN in ischemic patients with stroke were associated with the patient’s cognitive decline (Dacosta-Aguayo et al., 2015). The abnormalities of the resting-state functional connectivity in the DAN and the DMN are the effects of strokes on brain networks. Functional disruptions in these networks are linked to impairment of the patients’ cognitive functions, such as attention and motor functions. Thus, the decreased connectivity found in this study between the DAN and the DMN is consistent with these previous studies and reflects abnormal functional interactions among the DAN and the DMN.

Decreased internetwork connectivity among the VIS, DAN, and SMN was also observed in patients after stroke. The VIS is a subnetwork related to visual attention and receives top–down modulation from the frontal and parietal areas (Bressler et al., 2008). A previous study in patients with stroke showed abnormal activity in the visual occipital region, which is explained as a non-effective compensatory reorganization of motor deficits after stroke (Park et al., 2011). Motor deficits can also explain the disrupted coupling between the VIS and SMN in our results in patients with stroke. The limbic network is a system that includes the amygdala, the hippocampus, the cingulate gyrus, the hypothalamus, and the fornix. The hippocampus is essential for memory formation. The amygdala is thought to be important for processing emotions. The limbic system serves various functions, such as memory and emotions (Rolls, 2015). A previous study on poststroke depression demonstrated that the functional connections of the limbic system were reduced (Shi et al., 2017). The significant decrease in the FNC between the LIM and the DAN and between the LIM and the DMN may be the neuroimaging expression of their behavioral deficits after stroke. In the real world, patients with stroke rarely experience damage to only their motor function. A previous study revealed that emotional difficulties and memory deficits after stroke are common (Kneebone, 2016). All these physical or cognitive deficits and brain neuroimaging damage in ischemic stroke are induced by the deprivation of blood supply. The widely detected decrease in the FNC between networks may imply that stroke-induced alterations can occur in multiple functional systems. This view can also be supported by a previous graph theory study showing that the topological organization of the brain networks is altered in patients with stroke (Gratton et al., 2012).

### The recovery process in the functional network connectivity after a stroke

A stroke can induce a long-term motor or language disability, significantly reducing the quality of life of patients with stroke. Previous neuroimaging studies focused on cerebral reorganization following spontaneous motor recovery and demonstrated that all patients with stroke experience at least some predictable degree of functional motor recovery within the first few months after a stroke (Park et al., 2011; Carter et al., 2012b; Xu et al., 2014). This spontaneous functional recovery may be associated with functional and structural reorganization of the related subnetworks of the brain. In addition to spontaneous recovery, specific therapeutic intervention is another main method for determining the mechanism that underlies recovery after stroke. Previous studies demonstrated that intervention studies can provide further insights into the causal relationships between behavior and connectivity (Grefkes and Fink, 2011). In the present study, we used the ICA method to investigate the recovery process in brain functional patterns after a stroke. As expected, the depressed internetwork FNCs of the DAN, the DMN, the LIM, the VIS, and the SMN were enhanced from the first to the second time point in the patient group. No significant difference in internetwork FNCs was detected between the controls and the patients at the second time point. The results were consistent with previous findings, in that the intrinsic functional connectivity patterns in chronic patients with stroke showed plasticity with clinical intervention (Bajaj et al., 2015; Li et al., 2015a,^2017^; Zheng et al., 2016). Enhanced between-network functional connectivity may be induced by vascular repair after antiplatelet therapy or spontaneous recovery. Platelets can adhere to damaged blood vessels and agglutinate locally (Yardumian et al., 1986). Citicoline is a neuroprotective agent and a membrane phospholipid biosynthesis medium that can reduce cerebral vascular resistance, increase cerebral blood flow, improve brain function, and promote brain function recovery (Adibhatla, 2013). In the present study, patients with stroke were treated with antiplatelet therapy. The disruptions in the brain functions of patients with stroke were relieved at the second time point. Antiplatelet therapy may be the potential factor that induced this recovery. However, the present study included only one patient group with antiplatelet therapy. One patient group without any therapy should be included in this experiment to determine whether this recovery was induced by therapy alone. A previous study showed that spontaneous recovery could be seen in the first few months after a first-ever stroke (Ramsey et al., 2017). The stroke duration of all the patients in the present study falls within this time frame. Thus, the improvements in the FNC may also be due to spontaneous recovery. The restored FNC patterns of the present study only suggest an enhanced coupling between the cognitive networks. Future research should consider this question and improve our design to determine which factor is the reason for the present results.

Most previous studies on the longitudinal changes in stroke focused on the recovery of motor-related networks (James et al., 2009; Bajaj et al., 2015; Li et al., 2017). Our study is unique in our assessment of the functional reconfiguration of multiple networks in strokes with a longitudinal design. The reason is that cerebral stroke leave patients with significant psychological and physical impairments, such as motor, language, attention, and memory deficits. In the present study, we observed a significant increase in the functional coupling among the motor, visual, attention, and limbic subnetworks. Such changes in internetwork coupling in strokes can provide a new understanding of the ability for clinical recovery.

### Regression analyses

Notably, the FNC between the DAN and the LIM in patients with stroke showed a predictive regression with their posttreatment clinical scores. This regression result between the FNC score and the FMA score may suggest that the network coupling between the DAN and the LIM before treatment can reflect the recovery degree of the patients’ motor function ability. The present result was consistent with our expectation that the functional connectivity between networks in stroke would reflect the degree of the patients’ clinical recovery later. A previous study revealed that oscillatory brain activity during the rehabilitative intervention could be considered a biomarker of motor recovery in chronic stroke (Ray et al., 2020). The topological organization of the brain networks was altered in patients with stroke, and the abnormal nodal degree was positively correlated with the FMA score (Zhang et al., 2017). The topological properties of the brain can reflect motor impairment in patients with stroke (Li et al., 2021). The regression result between the FNC and the FMA is consistent with that of these previous studies. It implies that patients with stroke with few disruptions in the functional coupling between the DAN and the LIM would better recover their motor ability. Additionally, we observed that the functional coupling of the DAN and the LIM in patients with stroke showed a predictive regression with the NDS score at the second time point. Our previous study revealed that the FMA scores significantly and negatively correlated with the DNS scores in patients with stroke (Li et al., 2015b). This result may suggest that patients with stroke with few disruptions of their functional coupling between the DAN and the LIM would exhibit good neurological functional recovery. Thus, the functional coupling between the DAN and LIM can be regarded as a biomarker for predicting neurological function recovery in stroke. The functional coupling between the DAN and the LIM can be used to evaluate prognosis before stroke treatment.

Although significant neuroimaging-behavior relationships were detected in the present study, the FNC within the motor network did not show any predictive regression with the clinical behavioral scores. Previous studies revealed that the functional connectivity between the motor regions can predict the recovery of patients’ motor ability (Park et al., 2011; Rehme et al., 2011; Min et al., 2020; Li et al., 2021). The possible reason for this inconsistency may be that the motor components in the present study did not divide the bilateral regions. We used the mean signal of the bilateral motor regions to calculate the functional connectivity within the motor network. Our calculation of within-motor ICs could not detect the functional connectivity between the bilateral motor regions. Future studies should pay more attention to this question.

### Limitations

There were several potential limitations to this study. First, our analysis did not indicate increased FNC between subnetworks. This result is not consistent with that of a previous study that found both an increase and a decrease in the FNC among multiple networks (Zhao et al., 2018b). This inconsistency may be because the components used to select the template differed between the two studies. Future studies should reduce the template selection methods to obtain the final results. Second, the sample size was relatively small, and the acquisition time of our resting-state fMRI data was relatively short. This small sample size would limit the statistical power for detecting longitudinal changes in brain functional coupling. The short acquisition time may affect the quality of the imaging data. Future studies with a larger sample set are required to investigate the replicability of our findings. Third, the experimental design must be optimized. The present design cannot demonstrate whether the significant recovery was induced by the treatment or by spontaneous recovery. Thus, a group of patients with stroke who have not received any intervention should be included in the design. Finally, the patients in the current study had a wide range of duration of illness, which may affect our statistical results. Future studies would need to control the duration of illness of the patients.

## Conclusion

Using a longitudinal design, we investigated the changes in brain network patterns in patients with subcortical strokes. The patients with stroke demonstrated disruption in the FNC among multiple functional networks. The disrupted FNCs were restored to their normal level 1 month later. The results provided insights into the neural mechanisms of brain functional recovery after stroke. Moreover, the regression analyses revealed that the FNC between the DAN and LIM in patients with stroke showed predictive regression with their clinical scores 1 month later. Functional coupling between the DAN and LIM after stroke can be regarded as a biomarker for predicting the recovery of a patient’s motor function and the degree of neurological functional deficit. Overall, these findings may provide a novel opportunity to improve the prognostic ability of subcortical strokes. Future studies may benefit from including more information in the analysis to predict prognoses in stroke.

## Data availability statement

The original contributions presented in this study are included in the article/Supplementary material, further inquiries can be directed to the corresponding author YL, yxin-li@163.com.

## Ethics statement

The studies involving human participants were reviewed and approved by the Ethics Committee of Chengdu University of Traditional Chinese Medicine. The patients/participants provided their written informed consent to participate in this study.

## Author contributions

YL and PW designed the experiment, analyzed the data, and drafted this manuscript for the work. ZY and PW helped to acquire the clinical and fMRI data. JC helped to revise the manuscript critically for important intellectual content. YL, PW, and JC provided the financial support, reviewed the manuscript, and provided final approval for the manuscript to be published. All authors read and approved the final manuscript.

## Footnotes

1
http://www.fil.ion.ucl.ac.uk/spm

2
http://mialab.mrn.org/software/gift

3
https://surfer.nmr.mgh.harvard.edu/fswiki/CorticalParcellation_Yeo2011

## Supplementary material

Supplementary Figure 1 Lesion maps. **(A)** This figure demonstrates the locations of the lesions in the standard space of each patient included in this study. **(B)** This figure is the merged lesion map of all patients, in which all lesion maps were overlapped to produce this patient group lesion map. The right hemisphere lesions of two patients were flipped to the left hemisphere. The *n* values of the color bar in this map represent the number of patients. For example, one region showed yellow; in this region, the corresponding *n* value is 5, suggesting that five patients have lesions.

Supplementary Figure 2 Spatial maps. **(A)** The seven spatial maps of Yeo2011 reference networks, **(B)** spatial maps of the 19 independent components grouped into seven domains and compared to the Yeo 2011 reference networks.
