# Supplementary material for: Ability of an altered functional coupling between resting-state networks to predict behavioral outcomes in subcortical ischemic stroke: A longitudinal study
Source: Front Aging Neurosci. 2022 Sep 15;14:933567. doi: 10.3389/fnagi.2022.933567 (PMC9520312; doi:10.3389/fnagi.2022.933567)
Supplement: Supplementary file 1 [file Table_1.DOC]

**Supplementary Table 1. The lesion locations of each stroke patients**

| Patients | ***Lesion locations*** |
| --- | --- |
| Subj 1 | Left pons-centrum semiovale |
| Subj 2 | Left basal ganglia |
| Subj 3 | Left caudate nucleus and periventricular |
| Subj 4 | Right basal ganglia |
| Subj 5 | Left putamen  Left basal ganglia-centrum semiovale |
| Subj 6 |
| Subj 7 | Left basal ganglia-internal capsule |
| Subj 8 | Left basal ganglia and centrum semiovale |
| Subj 9 | Left basal ganglia |
| Subj 10 | Left basal ganglia-posterior horn of lateral ventricle |
| Subj 11 | Left centrum semiovale-periventricular |
| Subj 12 | Left periventricular |
| Subj 13 | Left thalamus-lenticular nucleus |
| Subj 14 | Right basal ganglia |
| Subj 15 | Left basal ganglia |
| Subj 16 | Left capsula externa-periventricular |
| Subj 17 | Left basal ganglia |
| Subj 18 | Left basal ganglia |
| Subj 19 | Left thalamus |
