# Supplementary figures and images for: Ability of an altered functional coupling between resting-state networks to predict behavioral outcomes in subcortical ischemic stroke: A longitudinal study
Source: Front Aging Neurosci. 2022 Sep 15;14:933567. doi: 10.3389/fnagi.2022.933567 (PMC9520312; doi:10.3389/fnagi.2022.933567)

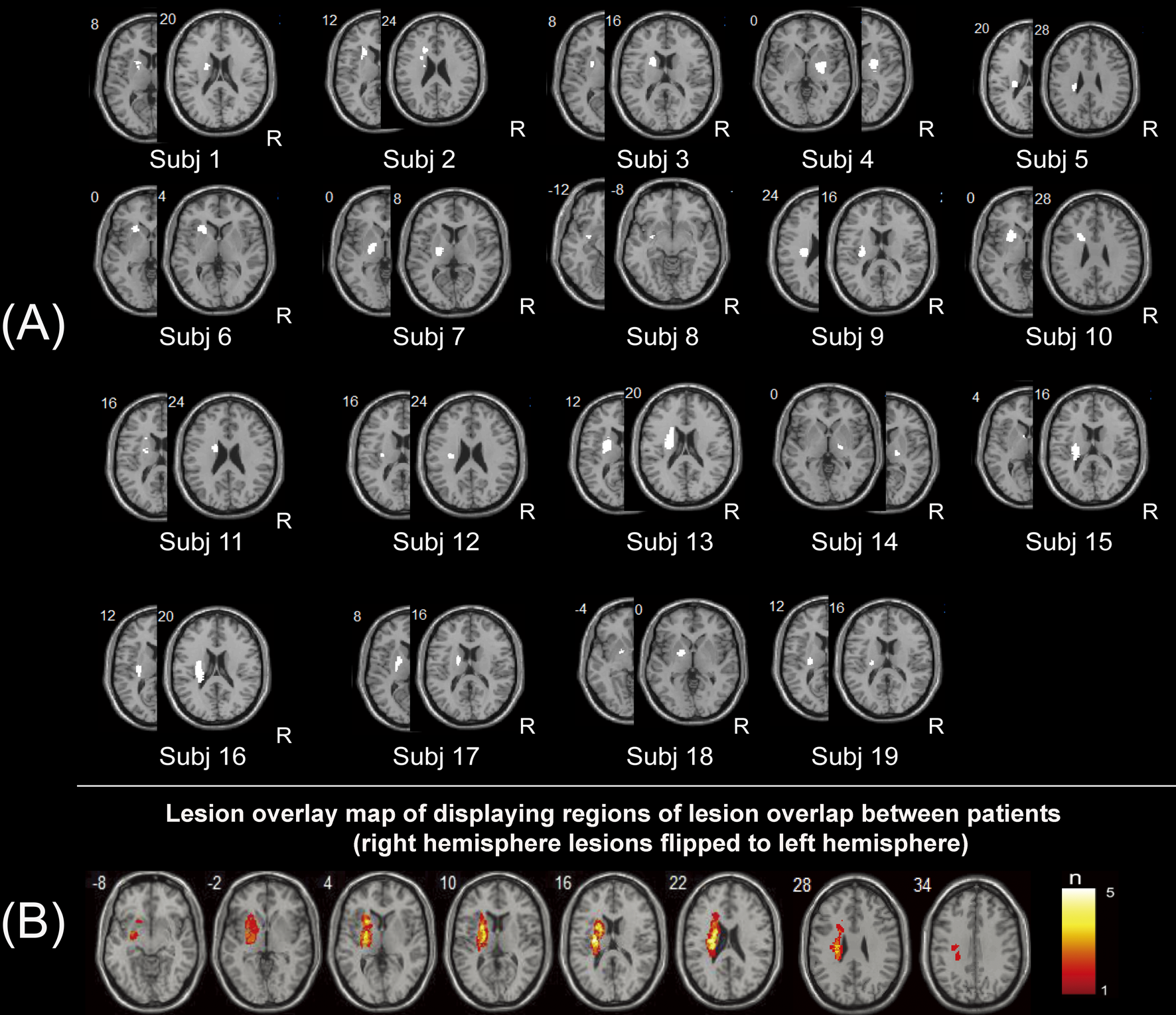

Supplement: Supplementary Figure 1 — Lesion maps. (A) This figure demonstrates the locations of the lesions in the standard space of each patient included in this study. (B) This figure is the merged lesion map of all patients, in which all lesion maps were overlapped to produce this patient group lesion map. The right hemisphere lesions of two patients were flipped to the left hemisphere. The n values of the color bar in this map represent the number of patients. For example, one region showed yellow; in this region, the corresponding n value is 5, suggesting that five patients have lesions. [file Image_1.TIF]

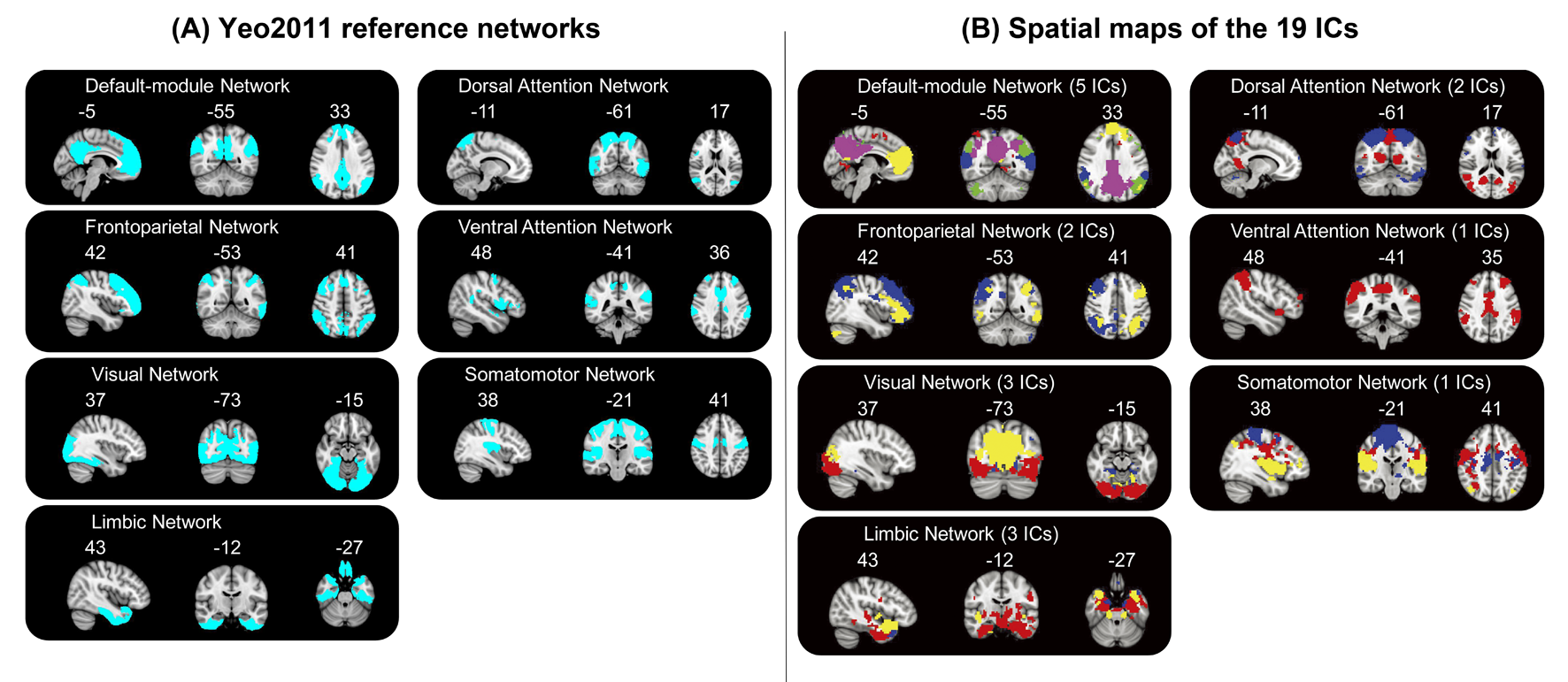

Supplement: Supplementary Figure 2 — Spatial maps. (A) The seven spatial maps of Yeo2011 reference networks, (B) spatial maps of the 19 independent components grouped into seven domains and compared to the Yeo 2011 reference networks. [file Image_2.TIF]
